# Occipital Neuralgia in Chiari I Malformation: Two Different Events or Two Different Faces of the Same Event?

**DOI:** 10.7759/cureus.1891

**Published:** 2017-11-29

**Authors:** Giacomo Tondo, Fabiola De Marchi, Daniela Mittino, Roberto Cantello

**Affiliations:** 1 Department of Translational Medicine, Neurology Unit, Azienda Ospedaliero Universitaria "Maggiore della Caritá", Novara

**Keywords:** occipital neuralgia, arnold chiari malformation

## Abstract

Occipital neuralgia (ON) is characterized by severe pain in the occipital region due to an irritation of the occipital nerves. Traumatic injuries, mass or vascular compression, and infective and inflammatory processes could cause ON. The dislocation of a nerve/muscle/tendon, as can happen in malformations such as the Chiari I malformation (CIM), also can be responsible. Usually, headaches associated with CIM and ON are distinguishable based on specific features of pain. However, the diagnosis is not easy in some cases, especially if a clear medical history cannot be accurately collected. Determining if the pain is related to ON rather than to CIM is important because the treatments may be different.

## Introduction

Occipital neuralgia (ON) is a headache characterized by severe, paroxysmal, stabbing, or shooting pain in the area of the greater, lesser, or third occipital nerve. Pain, unilateral or bilateral, originates in the occipital region; it may be associated with dysesthesia or allodynia due to pressure stimulation of the scalp. The distribution is due to the emergence of the greater occipital nerve over the C2 dermatomes [1]. It is described as a rare form of headache; nevertheless, in a recent paper, authors consider it as the third-most common headache syndrome, following migraines and tension-type headaches [2]. Other symptoms may be associated with the headache such as visual disturbances, nausea, dizziness, and tinnitus. Etiology includes trauma, compression due to vascular abnormalities and mass lesions, inflammatory and infective processes, fibrositis, myositis, or the dislocation of nerve/muscle/tendon structures due to arthrosis, sclerosis, osteolytic lesions, and Chiari I malformation (CIM) [3]. Treatment includes antidepressants, antiepileptics, infiltration of the nerve (anesthetic and corticosteroids), and botulinum toxin A. Diagnosis is clinical and is inferred from the response to local anesthetic block. We encountered a particularly complex case of ON, mainly because of difficulties in obtaining reliable clinical data.

## Case presentation

**Figure 1:**
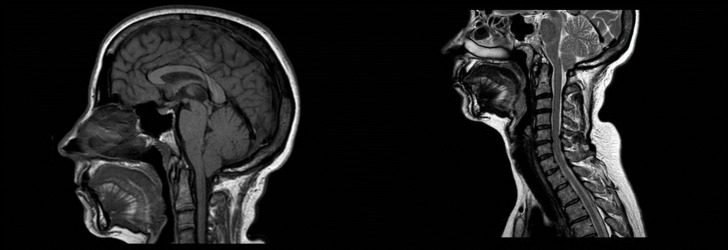
Brain MRI showing Chiari I malformation Brain MRI T1 (left) and T2 (right) weighted sagittal sequences show a Chiari I malformation. The tonsillar descent is about 8-8.5 mm. MRI: magnetic resonance imaging

A 44-year-old Togolese female presented to our headache center for refractory pain, which began three years ago. Her medical history was difficult to gather due to language problems. The patient complained of persistent occipital and cervical pain, with occasional paroxysmal attacks of stabbing pain accompanied by nausea and dizziness. No psychophysical comorbidity, no cigarette or alcohol use, or hormone therapies were described. A brain computed tomography (CT) scan with an angiographic study was normal. A neurologic examination revealed no abnormalities. A physical examination demonstrated paraspinal neck extensor tension. Amitriptyline (30 mg/day) was started, without benefit. An inconsistent response was obtained with nonsteroidal anti-inflammatory drugs. Six months later, a new therapeutic approach with flunarizine was attempted, but it was ineffective as well. Later, a prophylaxis with topiramate was initiated but was interrupted because of mood changes. At the same time, a brain magnetic resonance imaging (MRI) revealed a lowering of the cerebellar tonsils, compatible with CIM (Figure 1). However, the pain features were not compatible with CIM headaches because there was no postural change and the Valsalva or similar maneuvers did not exacerbate the symptoms. On neurological examination, we noticed a Tinel sign, on percussion of a region about 3 cm near the occipital protuberance, evocative of ON. Thus, the patient was referred to the pain clinic where she underwent treatment with two anesthetic blocks of the greater occipital nerve. This led to rapid and complete regression of the painful symptomatology.

## Discussion

ON is a debilitating headache localized in the occipital area. It can occur in cases of irritation, compression, or traction of the occipital nerves, as in the pathologic processes of the neck vessels, bones, muscles, or tendons. Association with CIM is rarely reported [4], but the prevalence of a simultaneous occurrence of the two diseases is unknown. In addition, the pathogenesis of headaches with CIM is unclear. CIM is reported to be related to dislocation/irritation of other cranial nerves when surgery can be healing [5-6]; however, we do not have a series report of the benefit of surgery in cases of ON in CIM. Moreover, it must be considered that both conditions could cause headaches in the same region, even with specific characteristics that are not always easily identified; this can make diagnosis a challenge.

In case of a suspicion of both CIM and ON, it is useful to consider the diseases as two different events. Indeed, until now, no conclusive data on a causative link between ON and CIM have been reported in the literature. For this reason, it is reasonable to try to treat ON with local anesthetic blocks before the surgical decompression of CIM, especially if the pain is stabbing or shooting, there is hypo/anesthesia or dysesthesia in the occipital region, and pain is not exacerbated by a cough, postural variation, or the Valsalva maneuver.

## Conclusions

In the present case, the language barrier and the finding of CIM on an MRI scan had originally confounded the origin of pain. This case emphasizes the importance of a conservative treatment approach in patients with clinical features of ON and an occasional finding of CIM. In these cases, treatment of ON with localized blocks may help patients with headaches, who will respond without surgery even in the presence of radiologic findings of CIM.
